# A call for action: integrating climate change into the medical school curriculum

**DOI:** 10.1007/s40037-019-00541-8

**Published:** 2019-10-09

**Authors:** Madelon L. Finkel

**Affiliations:** Weill Cornell Medicine, New York, NY USA

The world is experiencing unprecedented changes in the global climate system. The increase in the number of extreme weather events, including rising temperatures, extended heatwaves, hurricanes, flooding, droughts and wildfires, has been steadily increasing in every region of the world since the 1990s [1]. The global mean surface temperature has increased and atmospheric concentrations of carbon dioxide and other greenhouse gases (e.g., methane) have risen. Ice caps are melting; sea levels are rising. The 2018 Lancet Countdown report, which monitors the impacts of climate change on health, concluded that many of the global trends identified in its 2015 report (e.g., vulnerability to heat, vectorial capacity for disease, terrestrial and marine food security) have accelerated in terms of the impact of climate change on health [2]. The 2018 report ominously concludes that based on analysis of the data, climate change is ‘the biggest global health threat of the 21st century’.

The implications of extreme weather and climate events on the environment as well as on health status are apparent, with young children, the elderly and patients with comorbid conditions most vulnerable [3]. Twenty-three percent of global deaths and 22% of global disability adjusted life years (DALYs) were attributable to environmental risks in 2012, and nearly a quarter of global disease burden could be prevented by reducing environmental risks [4]. For example, coal burning power plants spew harmful levels of sulphur dioxide and particulate matter into the air and exposure to ambient air pollution and fine particulate matter have serious implications for respiratory diseases. Air pollution alone contributed to an estimated 6.5 million deaths in 2015 [5]. Heat stress exacerbates kidney and cardiovascular disease, among other conditions. As of 2017, 157 million more people were exposed to heat-related health risks than in 2000 [2]. Further, rising temperatures have implications for the transmission of important vector-borne and water-borne diseases, which have implications for the spread of many infectious diseases. Higher temperatures lead to an increase in mosquitos that transmit the viruses that cause Zika, chikungunya, yellow fever, and dengue. Even small changes in temperature and rainfall can have a significant effect on the spread of disease [2]. The World Health Organization (WHO) projects that between 2030 and 2050 there will be approximately 250,000 deaths annually due to climate change [6].

Climate change also contributes to reduced agricultural yields, which has an impact on food security and food availability. Changes in crop yields linked to climate change lead to food shortages, which can have substantial implications for nutritional status as well as for famines. In addition to its impact on physical health, climate change also affects mental health and wellbeing. As a result of climate change, there is a geographic displacement of populations, damage to property, and chronic stress, all of which can negatively affect mental health [2].

Some believe that climate change is too narrowly focused. The idea of Planetary Health was introduced in 2015 by The Rockefeller Foundation-Lancet Commission on Planetary Health [7]. Planetary health aims not only to investigate the effects of environmental change on human health, but also to study the political, economic, and social systems that govern those effects. In addition to assessing the impact on human health, planetary health also takes into account poverty, nutrition, gender equity, water and sanitation, energy, economic growth, industrialization, inequality, urbanization, human consumption and production, climate change, ocean health, land use, peace, and justice. As conceived, planetary health is a wide-ranging, interdisciplinary subject with a focus on addressing challenges to maintain and enhance human health in the face of increasingly harmful environmental trends.

What does this have to do with the medical school curriculum? There is a growing realization that as we prepare medical students to enter the profession, it is imperative that they graduate with an understanding of the implications of climate change/planetary health on the physical and mental health and well-being of their patients.

Given the mounting evidence that changes in the climate system have the potential to adversely affect human health, the medical school curriculum should incorporate curricula to reflect the health risks and harms associated with a changing climate. A 2013 survey found that 34–40% of each year’s graduating medical students between 2009 and 2013 believed that their instruction in environmental health was inadequate [8]. The implications of these findings take on added urgency as we learn more about the potential hazards of climate change on the environment and human health. Clearly, medical schools can and should do a better job of preparing medical students to be cognizant of and to understand the negative effects of climate change on human health.

The medical school curriculum is not static, evolving over the decades to reflect new ideas and new knowledge. An increasing number of medical schools are implementing changes to their curriculum to incorporate timely and current content. One-third of US medical schools report that they are planning to implement curricula changes in the near future while 31% are in the process of so doing. One fifth have already implemented changes within the past three years [9]. The potential impact of climate change on human health begs the importance of including the health impacts of climate change in the medical school curriculum. While some medical schools have introduced climate change modules focusing on the role of the environment as a risk factor for disease, [10] more widespread action is needed.

A decade ago an ad hoc Interagency Working Group on Climate Change and Health prepared a white paper outlining the human health effects of climate change [11]. While some progress has been made to mitigate the situation, much more remains to be done, including preparing medical students to understand the implications of climate change on the health and well-being of their patients.
